# Regulation of the global orthotist/prosthetist workforce, and what we might learn from allied health professions with international-level regulatory support: a narrative review

**DOI:** 10.1186/s12960-021-00625-9

**Published:** 2021-07-15

**Authors:** Leigh Clarke, Louise Puli, Emily Ridgewell, Michael P. Dillon, Sarah Anderson

**Affiliations:** 1 The Australian Orthotic Prosthetic Association Ltd, 2/1175 Toorak Road, Camberwell, Victoria 3124 Australia; 2 Discipline of Prosthetics and Orthotics, Department of Physiotherapy, Podiatry, Prosthetics and Orthotics, School of Allied Health, Human Services and Sports, La Trobe University, Melbourne, Victoria 3086 Australia

**Keywords:** Orthotist, Prosthetist, Assistive technology, Regulation, Certification, Credentialing, Practitioner, Standard, Workforce

## Abstract

**Background:**

By 2050, the global demand for orthotic and prosthetic services is expected to double. Unfortunately, the orthotic/prosthetic workforce is not well placed to meet this growing demand. Strengthening the regulation of orthotist/prosthetists will be key to meeting future workforce demands, however little is known about the extent of orthotist/prosthetist regulation nor the mechanisms through which regulation could best be strengthened. Fortunately, a number of allied health professions have international-level regulatory support that may serve as a model to strengthen regulation of the orthotic/prosthetic profession.

The aims of this study were to describe the national-level regulation of orthotist/prosthetists globally, and the international-level regulatory support provided to allied health professions.

**Method:**

Two environmental scans benchmarked the national-level regulation of the orthotist/prosthetist workforce, and the regulatory support provided by international allied health professional bodies using a set of nine core practitioner standards (core standards) including: *Minimum Training/Education*, *Entry-level Competency Standards*, *Scope of Practice*, *Code of Conduct and/or Ethics*, *Course Accreditation*, *Continuing Professional Development*, *Language Standard*, *Recency of Practice*, and *Return-to-Practice*. Each identified country was categorised by income status (i.e. High-, Upper-Middle-, Lower-Middle-, and Low-Income countries).

**Results:**

Some degree of regulation of the orthotist/prosthetist workforce was identified in 30 (15%) of the world’s 197 countries. All core standards were present in 6 of these countries. Countries of higher economic status had more core standards in place than countries of lower economic status. International-level professional bodies were identified for 14 of 20 allied health professions. International bodies for the physical therapy (8 core standards) and occupational therapy (5 core standards) professions provided regulatory support to help national associations meet most of the core standards.

**Conclusion:**

Given the small proportion of countries that have national practitioner regulatory standards in place, most orthotist/prosthetists are working under little-to-no regulation. This presents an opportunity to develop rigorous national-level regulation that can support workforce growth to meet future workforce demands. Given the financial and expertise barriers that hinder the development of a more regulated orthotist/prosthetist workforce, particularly for Low- and Lower-Middle-Income countries, we recommend the establishment of an international professional body with the express purpose to support national-level regulation of orthotist/prosthetists, and thereby build the regulatory capacity of national orthotic/prosthetic associations.

## Background

Globally, only 1 in 10 people have access to the assistive technology they need [1]. Assistive technology is an umbrella term describing the application of systems and services related to assistive products. Assistive products—including orthoses (splints/braces) and prostheses (artificial limbs)—promote functioning and independence that allows participation in education, employment and activities that bring joy and meaning to life. Orthoses and prostheses are required by a diverse range of people across the lifespan including people living with: limb loss, diabetic foot ulceration, post-stroke, cerebral palsy, scoliosis, post-polio syndrome, and lymphoedema, as well as management post-acute injury and surgery, as examples.

The global demand for orthotic/prosthetic services is expected to double by 2050—particularly in Low- and Lower-Middle-Income settings—as the world’s population ages, and the prevalence of non-communicable diseases grows [2]. Given current numbers of orthotist/prosthetists per 100 000 population are low [3, 4], significant growth of an appropriately qualified and competent workforce [5–7] will be required to meet the future global demand for orthotic/prosthetic services.

Practitioner regulation provides a mechanism to support workforce growth whilst ensuring practitioners have the requisite competencies to provide safe and effective services. This is achieved through setting and monitoring compliance with education standards (e.g., *Course Accreditation* standards), entry to practice standards (e.g., *Competency Standards*), ongoing practice standards (e.g., *Continuing Professional Development)* and behavioural standards (e.g., *Code of Conduct*), as examples.

When practitioners are regulated, government agencies and funding bodies can better understand a profession’s contribution to health care which, when enshrined in policy, can establish formal recognition of a profession [8]. When a profession is formally recognised, government agencies are more likely to establish defined roles and career pathways within the profession (e.g., within government hospitals) [2], thus providing access to higher salaries, greater employment stability and future promotion opportunities [2, 9, 10] that support workforce growth [11]. Formal recognition may also lead to improved funding and subsidies for services.

Presently, little is known about the extent of regulation of orthotist/prosthetists globally, nor the regulatory mechanisms in place. In a handful of countries, including the United Kingdom, orthotist/prosthetists are government-regulated. However, for many countries, the orthotist/prosthetist workforce is small, under-resourced [12], and lacks government recognition as a health care provider [2, 7, 13]. In these countries, regulation is often the responsibility of national associations who autonomously establish and enforce the core regulatory standards [14–16]; a profession-led regulatory model, known as self-regulation [17, 18]. For small and under-represented professions, such as orthotist/prosthetists, self-regulation has many benefits [17] (e.g., minimised regulatory burden and administrative cost) [2, 16, 19] given the standards applied are appropriate to each profession’s needs and context [2]. This is particularly valuable in Low- and Lower-Middle-Income countries where the cost and administration of complex and legislated regulation is unlikely to be prioritised, further impeding workforce growth [19].

There are significant barriers to establishing self-regulation, particularly for small professions such as orthotist/prosthetists, given the limited financial resources and expertise available [16]. For national associations, many of these barriers could be overcome through regulatory support provided by an international professional body [15] as recommended by the World Health Organization (WHO) Workforce Report 2030 [20]. Some allied health professions (e.g., physiotherapy, occupational therapy) are supported through international professional bodies and may serve as a model for the orthotic/prosthetic profession; particularly if the sort of regulatory support provided by these international professional bodies could be clearly described, and used to develop an international professional body with the express purpose of enhancing national-level regulation of the orthotic/prosthetic profession [16].

Given that little is known about the extent of national-level regulation of orthotist/prosthetists, and the opportunity to support regulatory efforts through the formation of an international professional body focused on supporting national-level regulation, the aims of this study were to describe the:

1. national-level regulation of orthotist/prosthetists globally;
2. regulatory support provided by existing international-level allied health professional bodies to their national member associations.

While an early version of this research was published as part of the World Health Organization’s Global Report on Assistive Technology (GReAT) Consultation 2019 [21], the intent of this article is to translate this work to a wider audience given the paucity of research focused on regulation of the global orthotist/prosthetist workforce and the importance of this research to strengthening regulation that helps improve access to assistive technology.

## Method

The first stage of this study was to define a core set of practitioner regulatory standards (i.e. core standards) that could be used to benchmark:

- Part 1—national-level regulation of orthotist/prosthetists globally;
- Part 2—regulatory support provided by existing international-level allied health professional bodies, to their national associations.

### Definition of the core set of practitioner standards

In the absence of international agreement on the definition of ‘core’ practitioner regulatory standards, the standards defined by the Australian Health Practitioner Regulation Agency (AHPRA) [22] were used. AHPRA is the body responsible for Government regulation of medical and allied health professions in Australia and is recognised as a leading health workforce regulator by the WHO Collaborating Centre [15, 23].

There are 11 standards applied to the AHPRA regulated professions that are defined through legislation [24] and profession-specific registration requirements [25]. Of the 11 standards, 9 were included in the core standards (Table 1). Criminal history declarations and professional indemnity insurance were excluded given their setting-specific nature [22].

**Table 1:** Nine core health practitioner regulatory standards

Regulatory standard	Definition and purpose of regulatory standard
Minimum Training/Education	The minimum training and/or education level required for individuals to practice in the profession This standard communicates the minimum training requirements to practice, to the community, external stakeholders and training institutions
Entry-level Competency Standards	An outline of the minimum skills and knowledge that must be demonstrated by individuals to practice in the profession This is an assessable standard which is used by training institutions to determine the required training content. It is also used by authorities responsible for assessing competency to determine whether international practitioners can practice in the profession
Scope of Practice	A guidance document which describes the role and activities a practitioner is permitted to undertake based on their training and qualifications This guidance is used to ensure the community and external stakeholders are aware of the boundaries of practice for an profession. It is commonly used to promote the services of a profession, but also to support disciplinary processes as working within one’s scope of practice is typically a component of a code of conduct
Code of Conduct and/or Ethics	Describes the conduct expected of practitioners in providing a health service and/or the values and principles required to be upheld by a practitioner This code defines the behavioural and ethical expectations to which the community can hold a practitioner to account. The code is commonly used in complaint and disciplinary processes and therefore each component must be assessable
Course Accreditation	A standard that training institutions must meet to be accredited by the national body for the education of practitioners Course accreditation ensures that training programmes deliver practitioner education in line with the competency standards and scope of practice for the profession and therefore ensure the future workforce meets the needs of the population and the health system
Continuing Professional Development	Describes the minimum requirement for ongoing education, typically on an annual basis This standard ensures that practitioner’s education journey is life-long and appropriate to their area of practice. It provides protection of the public by ensuring practitioners knowledge and skills are current
Language Standard	National language standards define the level to which a practitioner can adequately speak the primary language of the country This standard supports consumer safety by ensuring services are delivered by practitioners who can sufficiently communicate, or where language is a barrier, that alternative safeguards, such as translators are used
Recency-of-Practice	Describes the minimum amount of time that a practitioner can be absent from The workforce before a return to practice programme must be completed prior to workforce re-entry This standard provides protection to the public by ensuring services are delivered by practitioners with current knowledge and skills
Return-to-Practice	Describes the pathway to return to the workforce after a period of absenteeism from the workforce, as defined by the recency-of-practice standard This standard ensures that practitioners are sufficiently current before returning to practice, thereby supporting retention in the workforce, whilst simultaneously ensuring services are delivered by practitioners with current knowledge and skills

### Part 1—environmental scan of national-level regulation of orthotist/prosthetists globally

For the purpose of this environmental scan, the following definitions were applied:

- ‘National association’: an organisation established by members of a profession that acts nationally in the interests of the profession [15];
- ‘Regulator’, including ‘Government regulator’: an organisation that controls the practice of practitioners by setting standards, maintaining a register of practitioners, and taking action when registered practitioners do not meet the standards [26].

To help ensure that global efforts to regulate the orthotist/prosthetist profession were captured, these definitions were applied in two search strategies executed in June 2019. First, the *Health Regulation Worldwide database* [27] was searched to identify countries with a listed orthotic/prosthetic national association and/or regulator, using the database’s pre-defined search term for the profession, ‘*Prosthetists & Orthotists’*. Secondly, recognising that some orthotic/prosthetic national associations may be emerging (i.e. informal and/or still in their infancy) and therefore not recorded on the *Health Regulation Worldwide database*, additional searches using *Google* and social media sites were conducted for all countries not identified in the first search. The search strategy used country name in combination with the following terms and their synonyms:

- National Alliance, Association, Society, Union, Council
- Orthotist/prosthetist, orthotics/prosthetics (used in combination with conjunctions, e.g., orthotist AND prosthetist); orthopaedic technologist, orthopaedic technician.

Countries were included where the national association and/or regulator had publicly available information in English and was considered to be active based on web content, active social media, or responded to an email request for clarification. Where no national association and/or regulator was identified, no further investigation was conducted, such as investigation of system or organisational-level credentialing programmes. For all countries meeting the inclusion criteria, an environmental scan was conducted to determine the extent to which the orthotist/prosthetist profession was regulated. This included searching for the presence of the nine core standards in all publicly available information by one investigator (LP) with review by a second investigator (LC), or a request for clarification by email.

Given that Low- and Lower-Middle- Income Countries [28, 29] often lack the resources needed to establish professional regulation [16, 18], each identified country was categorised by income status (i.e. High-, Upper-Middle-, Lower-Middle-, and Low-Income countries) according to the World Bank classification [30, 31]. Therefore, the following data were extracted: country, name of association and/or government regulator, income status, model of existing regulation (i.e. self- or government-regulated), and whether each core standard was ‘present’ or ‘not present’.

### Part 2—environmental scan of regulatory support provided by existing international-level allied health professional bodies, to their national associations

For the purpose of this environmental scan the following definitions were applied:

- ‘Allied health’ profession: those professions holding full membership of *Allied Health Professions Australia* (AHPA); the peak body for allied health professions in Australia [32], in lieu of an internationally recognised definition.
- ‘International-level professional body’: an organisation with an international purpose related to a specific profession where membership consisted of independent national associations or entities. Given this definition, international-level professional bodies with individual practitioner memberships (e.g., International Expressive Art Therapy Association) or a multi-disciplinary purpose (e.g., the International Society of Prosthetics and Orthotics, ISPO) were excluded.

To ensure that international-level professional bodies providing regulatory support to allied health professions were captured, these definitions were applied in two search strategies executed in June 2019. First, the *Health Regulation Worldwide database* [27] was searched to identify allied health profession with a listed international-level professional body*.* Recognising that some professions may have emerging international bodies, a second search using *Google* was conducted for all allied health professions that were not identified in the first search. This search used profession name (e.g., physiotherapy) combined with the following search terms and their synonyms:

- World, Global, International
- Council, Federation, Confederation, Alliance, Association, Society, Union.

For each identified international professional body, an environmental scan of publicly available information, published in English, was conducted. The following data were extracted by one investigator (LC): organisation name, number of member organisations (i.e. national associations), whether support was provided for each core standard (defined as ‘in place’ or ‘absent’).

### Data analysis

Data from Part 1 described the number of countries with orthotist/prosthetist regulation, and the extent to which national-level regulation occurred. Data from Part 2 described the number of allied health professions with an international-level professional body, and the extent to which the body provided regulatory support to its member associations. Names of countries and national associations were de-identified given the aim was to provide a summary of global orthotist/prosthetist regulation, rather than rank countries based on implementation of the core standards.

## Results

### Part 1—environmental scan of national-level regulation of the orthotic/prosthetic profession globally

#### Countries with national associations and/or regulator

**Fig. 1:**
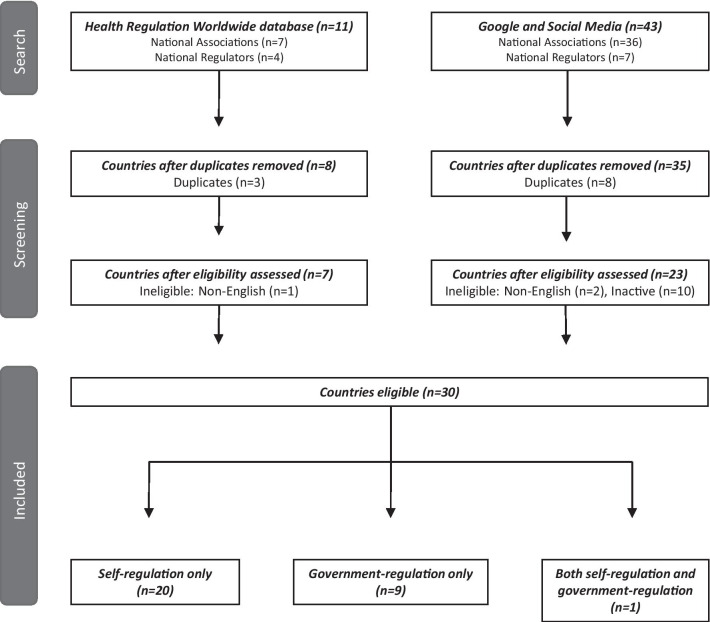
Flowchart of environmental scan search yield for Part 1—national-level regulation of the orthotist/prosthetist profession globally

Given the results from the two search strategies (*n* = 54), and removal of duplicate (*n* = 11) and ineligible records (non-English language *n* = 3; inactive *n* = 10), a total of 30 countries were included in the national-level scan (Fig. 1).

#### Presence of core standards for national-level regulation of orthotist/prosthetists

Of the 30 countries that met the inclusion criteria, 6 countries had all nine core standards in place (Table 2). The remaining countries had between 4 and 8 core standards (*n* = 13), while the others had 3 or less core standards in place (*n* = 11) (Table 2).

**Table 2:** Presence of nine core standards for orthotist/prosthetist regulation by country and income category (High Income, Upper-Middle Income, Lower-Middle Income and Low Income)

	Presence of Government Regulation	Presence of Core Standards									Number of Core Standards (Total)
		Minimum Training/Education	Entry-level Competency Standards	Scope of Practice	Code of Conduct	Course Accreditation	Continuing Professional Development	Language Standard	Recency-of-Practice	Return-to-Practice	
High-Income country *n* = 14 (47%)											
1	–	X	X	X	X	X	X	X	X	X	9
2	–	X	X	X	X	X	X	X	X	X	9
3	X	X	X	X	X	X	X	X	X	X	9
4*	X	X	X	X	X	X	–	X	X	–	7
5	X	X	X	X	X	X	X	X	X	X	9
6	–	–	–	–	X	n/a	X	–	–	–	2
7	X	X	–	–	–	n/a	–	–	–	–	1
8*	X^	X	X	X	X	X	X	X	X	X	9
9*	–	–	–	–	–	–	–	–	–	–	0
10*	X	X	X	X	X	X	–	X	X	–	7
11*	–	X	–	–	–	–	–	–	–	–	1
12	X	X	X	X	X	X	X	X	X	X	9
13*	–	X	X	X	X	X	–	X	X	–	7
14*	–	X	X	X	X	X	X	X	–	–	7
Upper-Middle-Income country *n* = 4 (13%)											
15*	–	X	X	X	X	X	–	–	X	X	7
16*	–	X	X	–	X	X	X	–	X	–	6
17*	–	X	X	X	X	X	X	X	X	–	8
18*	–	X	X	–	–	X	X	X	X	X	7
Lower-Middle-Income country *n* = 5 (17%)											
19*	–	X	X	X	–	X	–	X	–	–	5
20	X	X	–	–	–	X	X	–	–	–	3
21*	–	X	–	–	–	–	–	–	–	–	1
22*	–	X	X	–	X	X	–	–	X	X	6
23*	–	X	X	–	X	–	–	–	–	–	3
Low-Income country n = 7 (23%)											
24*	–	–	–	–	–	n/a	X	X	–	X	3
25*	–	–	–	–	–	n/a	–	–	–	–	0
26*	X	X	X	–	X	X	X	–	–	–	5
27*	X	X	X	–	X	X	–	X	–	–	5
28*	–	–	–	–	–	n/a	–	–	–	–	0
29*	–	X	–	–	–	n/a	–	–	–	–	1
30*	–	X	X	X	X	n/a	–	X	X	X	7
Total (%)	10 (33%)	25 (83%)	20 (67%)	14 (47%)	19 (63%)	19 (63%)	14 (47%)	16 (53%)	15 (50%)	11 (37%)	

X = standard in place;–= standard absent

*represents those countries where email contact was made with the association/regulatory to clarify the scan data (*n* = 22)

^ represents one country whereby specific states/regions had a government regulator

n/a signifies the absence of an in-country training programme

High- and Upper-Middle-Income countries had more core standards in place than Lower-Middle- and Low-Income countries (Table 2). For example, only High-Income countries had all nine core standards in place. In contrast, no Lower-Middle and Low-Income countries had all nine core standards in place. Lower-Middle and Low-Income countries commonly had 3 or less core standards in place (Table 2). Of the 11 countries with three or fewer standards, nearly two-thirds (64%) were Lower-Middle and Low-Income countries (Table 2).

The most commonly implemented core standard was Minimum Training/Education; present in 25 countries (83%). The Course Accreditation core standard was present in 19 countries (63%), which after adjusting for the seven countries without in-country training programmes, gives a rate of 83% (19/23). Four core standards—Code of Conduct (*n* = 19), Entry-level Competency Standards (*n* = 20), Language Standard (*n* = 16) and Recency-of-practice (*n* = 15)—were present in at least 50% of the countries (Table 2).

### Regulatory model

The majority of countries had adopted a self-regulation model for orthotist/prosthetists (*n* = 20; 67%). Nine countries had government regulation of orthotist/prosthetists. One country had both regulatory models in place whereby specific states/regions had a government regulator (Fig. 1).

### Part 2—environmental scan of regulatory support provided by international-level allied health professional bodies, to their national associations

#### Allied health professions with international professional bodies

Fourteen allied health professions (70%) had an international body providing some regulatory support to its member associations (Table 3). The 6 professions without an international professional body included: genetic councillors, creative art therapists, perfusionists, rehabilitation counsellors, exercise physiologists, and orthotist/prosthetists. There were no exclusions from the search results due to the English language inclusion criteria.

**Table 3:** International allied health professional bodies and presence of support provided for implementation of nine core regulatory standards

Profession, name of international body and number of member organisations (n)	Minimum Training/Education	Entry-Level Competency Standards	Scope of Practice	Code of Conduct	Course Accreditation	Continuing Professional Development	Language Standard	Recency-of-Practice	Return-to-Practice
**Physical therapy** World Confederation for Physical Therapy (*n* = 120)	X	X	X	X	X	X	X	X	–
**Occupational therapy** World Federation of Occupational Therapists (*n* = 101)	X	X	X	X	X	–	–	–	–
**Social work** International Federation of Social Workers (*n* = 123)	X	–	X	X	–	–	X	–	–
**Speech and language therapy (inc audiology)** International Association of Logopedics and Phoniatrics (*n* = 63)	X	–	X	X	X	–	–	–	–
**Dietetics** International Confederation of Dietetic Associations (*n* = 50)	X	X	–	X	–	–	–	–	–
**Medical imaging** International Society of Radiographers and Radiological Technologists (*n* = 80)	X	–	–	–	X	–	X	–	–
**Optometry** World Council of Optometry (*n* = unknown)	–	X	X	–	–	–	X	–	–
**Osteopathy** Osteopathic International Alliance (*n* = 10)	X	X	X	–	–	–	–	–	–
**Psychology** International Union of Psychological Science (*n* = 89)	X	X	–	X	–	–	–	–	–
**Music therapy** World Federation of Music Therapy (*n* = 26)	X	–	–	X	–	–	–	–	–
**Orthoptics** International Orthoptic Association (*n* = 16)	X	–	–	X	–	–	–	–	–
**Chiropractic** World Federation of Chiropractic (*n* = 88)	X	–	–	–	–	–	–	–	–
**Audiology** International Society of Audiology (*n* = unknown)	–	–	–	–	–	–	–	–	–
**Podiatry** International Federation of Podiatrists (*n* = 31)	–	–	–	–	–	–	–	–	–

#### Provision of regulatory support

No allied health profession had an international body providing regulatory support for all nine core standards (Table 3). While the physical therapy profession received support for the highest number of core standards (n = 8) through the World Confederation for Physical Therapy (WCPT) (now known as World Physiotherapy), most professions received regulatory support for less than half of the core standards (Table 3).

The core standard most often supported was Minimum Training/Education, with more than 75% (*n* = 11) of international bodies providing some regulatory support. A Code of Conduct (*n* = 8, 57%), Entry-level Competency Standards (*n* = 6, 43%) and Scope of Practice (*n* = 6, 43%) were supported by approximately half of the international bodies. No support was provided for the Return-to-Practice standard (Table 3).

## Discussion

This environmental scan highlighted that only a small proportion of the global orthotic/prosthetic profession was regulated. Only 3% (*n* = 6) of the world’s 197 countries [27, 33] had all nine core standards in place. Another 7% (*n* = 13) of countries had between 4 and 8 core standards in place. While the remaining 6% (*n* = 11) of countries have few core standards in place and may be working toward greater regulation, it is reasonable to conclude that there is little-to-no regulation of the orthotic/prosthetic profession in the vast majority of countries around the world; confirming observations made by at least one other author [8].

Practitioner regulation can play an important role in improving access to orthotic/prosthetic services [2, 20, 34]. Given self-regulation is currently the dominant regulatory model for orthotist/prosthetists, and represents a right-touch approach, there is an urgent need to provide regulatory support to national associations working towards the regulation of orthotist/prosthetists around the world.

While there is little doubt about the benefits of strengthened regulation and the opportunity afforded by regulatory support, there are real implementation challenges. As such, a detailed account is offered of the recent experience of the Australian Orthotic Prosthetic Association (AOPA), after developing and implementing all nine core standards within a self-regulatory framework. This first-hand account is provided to highlight the implementation challenges given the financial and expertise barriers faced by a small and largely unrecognised profession, and the longer term benefits of self-regulation, including the impact of improved regulation on national-level policy (i.e. Government and funders) and workforce growth.

### Exploration of orthotist/prosthetist regulation: a national example

Of the 6 countries with all core standards in place, three employed a self-regulatory model overseen by a national association. Australia is one of these countries where orthotist/prosthetists are self-regulated by AOPA.

AOPA was first established in 1975. It was not until 2013 that the process to establish self-regulation of orthotist/prosthetists began; a process that took 5 years to implement all nine core standards.

The experience of implementing self-regulation of the orthotic/prosthetic profession in Australia was not without its challenges. Given the small membership (about 270 members in 2013), AOPA had limited financial resources and a lack of expertise in practitioner regulation. Fortunately, AOPA was successful in applying for government grants that allowed the employment of staff to develop the first core standards: AOPA’s Entry-level Competency Standards [35, 36], Course Accreditation Standards [37] and the associated Entry-to-Practice assessment processes. AOPA also received support from the National Alliance of Self-Regulating Health Professions (NASRHP), a peak organisation that supports self-regulation of the non-Government regulated allied health professions in Australia, including: speech pathology, dietetics, social work, and orthotics/prosthetics, as examples [38]. NASHRP promotes peer-to-peer mentoring between associations, which allowed shared learning from our allied health counterparts. It would not have been possible for AOPA to achieve full self-regulation without the support from government grants and NASRHP.

Many benefits were observed as a result of establishing regulation of orthotist/prosthetists in Australia. First, funding bodies including the Australian Department of Veterans’ Affairs and private health insurers had increased confidence in the profession, given regulated practitioners now met the required core standards. This resulted in greater recognition of orthotist/prosthetists [39] and subsequent changes to funding policies; for example, orthotist/prosthetists were allowed to deliver autonomous services without medical oversight which streamlined access to funded services for users. Second, establishing regulation of orthotist/prosthetists in Australia resulted in Government policy change that assisted workforce growth by facilitating entry to the profession of experienced internationally qualified orthotist/prosthetists. For example, in 2017 the Australian Government Department of Immigration reviewed the orthotist/prosthetist workforce profile and identified a critical workforce shortage. The orthotist/prosthetist profession was added to the Department of Immigration’s Skilled Occupations List [40], thereby permitting skilled migration of internationally qualified orthotist/prosthetists, subject to meeting the core Entry-to-Practice standards, with AOPA legislated as the assessing authority [40].

### Exploration of regulatory support: an international example

AOPA received regulatory support in the form of expertise and resources from NASRHP; a body with the express purpose to support self-regulation of the non-government regulated allied health professions in Australia. Bodies providing similar regulatory support at an international level are already in place across nearly two-thirds of all allied health professions. A benefit of offering regulatory support, rather than regulation oversight, is that this allows each country to implement a regulation framework appropriate to the local setting [13, 41], without being unnecessarily burdensome [19] or creating barriers that limit access [13, 16]. An early indication of the potential impact of such regulatory support is evident in the results of this study; the most common core standard implemented for orthotist/prosthetists was Course Accreditation which has been supported through the ISPO Accreditation Pathway [42] and endorsed by the WHO through published orthotic/prosthetic practice standards [2].

The WCPT provides an example of an international professional body providing comprehensive regulatory support to its 120 member associations, across 8 of the nine core standards. The WCPT clearly defines the role that national practitioner regulation plays in building confidence in the profession and ensuring the delivery of safe and effective services:

“WCPT encourages member organisations to work towards a system of regulation that focuses on the public interest. Such a system will promote trust and confidence in the profession” [43]

“. . . in some countries, the profession is regulated by physical therapists meeting membership criteria for the professional organisation . . . In many cases, effective regulation can be achieved by embedding standards of professional education, performance, conduct and competence within the system of regulation. These standards, together with mechanisms to monitor and foster practitioner compliance and manage non-compliance, provide the means by which the profession can protect the public interest [43].”

Other international professional bodies demonstrate the value of regulatory support through formal mentoring programmes to support countries with emerging regulation. For example, the International Federation of Social Workers has a mentor programme, known as “twinning” [44, 45] where a mature national association with well-developed regulation provides support to an emerging national association [46].

“The Moroccan association AMAS (Association Marocaine des Assistants et Assistantes Sociales) is still young, just like social work education in Morocco . . . Now both social work associations have decided to start twinning; to inspire each other. The still young association can be supported from the Netherlands, and Dutch social workers can learn from work in different circumstances and how to set up initiatives were [sic] governmental support is lacking or only providing a part of what is needed [45].”

The barriers to self-regulation experienced in Australia are likely universal, though exacerbated for those in Lower-Middle- and Low-Income countries due to the relative immaturity of health services and poor recognition of orthotist/prosthetists [2, 47]. The results suggest that Lower-Middle- and Low-Income countries had fewer standards in place than High- and Upper-Middle- Income countries and as such, these countries would particularly benefit from regulatory support provided by an international-level professional body.

## Recommendations

Orthotist/prosthetist regulation builds workforce capacity, stakeholder confidence and increases access to safe and effective services. On this basis, we recommend the following to the international orthotic/prosthetic community and international agencies seeking to increase global access to orthotic/prosthetic services:

- All countries should seek to establish a national orthotic/prosthetic association, regardless of workforce size or resources (e.g., financial or expertise);
- Where government regulation of orthotist/prosthetists is absent, national associations should work toward a self-regulatory framework;
- Where existing national orthotic/prosthetic associations already operate in a self-regulatory framework, all core standards should be developed and implemented.

Given it will not be possible to achieve the above recommendations without extensive regulatory support, we also recommend:

- The establishment of an international professional body for the orthotic/prosthetic profession with the express purpose to support national-level regulation of orthotist/prosthetists across all core standards.

Pertaining to this international professional body for orthotist/prosthetists, we recommend:

- Global stakeholder consultation to guide the establishment of the international body, including but not limited to orthotist/prosthetists, government representatives, policy makers, users of services and other allied health professionals;
- Limiting membership of the international body to national associations for the orthotic/prosthetic profession; acknowledging that many national associations may not currently be legal entities, but can demonstrate progress towards formalisation;
- Provide leadership to establish expectations for orthotist/prosthetist regulation by developing position statements for each core standard (e.g., position statement stating the conduct and ethical behaviour expected of orthotist/prosthetists and the requirement for a national code of conduct to be in place);
- Remove expertise barriers that hinder *development* of the core standards by providing example documents for each standard that supports adaptation for the national setting (e.g., example code of conduct for orthotist/prosthetists that is generic across contexts);
- Remove expertise barriers that hinder *implementation* of the core standards by providing guidance documents describing the required policies and procedures—including exemplars—that underpin the standards (e.g., implementation of a national code of conduct requires policies and procedures pertaining to complaint handling, disciplinary actions and formation of a committee to oversee such complaints effectively);
- Establish formalised programmes that support peer-to-peer relationships where national associations with expertise in standard implementation can support emerging associations (e.g., a “twinning” programme);
- Remove financial barriers to establishing regulation by creating opportunities for financial support (e.g., grant funding) for national associations through engagement with international organisations (e.g., WHO and ATscale) and national stakeholders (e.g., national Governments and Ministries of Health) who wish to support the development of effective national-level regulation;
- Establish a register of orthotic/prosthetic national associations for the purpose of communicating regulatory efforts to the international community, including collection of global orthotist/prosthetist workforce and regulation data in all 6 of the recognised United Nations languages.

These recommendations seek to outline key features of an international body with the express purpose to support national-level regulation of orthotist/prosthetists globally. A thoughtful approach will be required to build such an international organisation, whereby those with demonstrable expertise can provide leadership. The required expertise and experience likely already exist amongst the 6 countries that have all core standards in place and therefore these organisations are well placed to guide the next steps. This approach to establish an international body by leveraging expertise within existing national associations has been successfully implemented in the assistive technology sector, through the recent establishment of the Global Alliance of Assistive Technology Organisations (GAATO) by a founding group of ten national assistive technology associations [48].

## Limitations

This environmental scan may under-represent the number of core standards in place, given our inclusion criteria required publicly available information published in English, or successful email contact with an association/regulator to clarify details (Table 2).

Additionally, countries with regulation in place may have been inadvertently excluded from the scan, because we were unable to establish eligibility given the publically available information was not in English, and we were unable to obtain clarification from the association/regulator via email.

For example, 13 countries with an identified association/regulator were excluded from the scan (Fig. 1), 3 countries were excluded based on a non-English website and unsuccessful contact with the association/regulator; and 10 countries were excluded because an association/regulator identified via social media could not be confirmed as active (e.g., no website and/or no social media content). The under-representation due to the English language exclusion (*n* = 3) and lack of activity (*n* = 10) is likely to be small, given countries with substantial practitioner regulation will likely be listed on the *Health Regulation Worldwide database.*

Further, some readers may be surprised that organisations such as ISPO were excluded from the international-level environmental scan. For clarity, the ISPO does not meet the two components of the inclusion criteria of an international professional body; that is, having a purpose related to a specific profession (e.g., orthotist/prosthetists), and a membership consisting of independent business entities (e.g., national associations for orthotist/prosthetists).

Given the core standards were defined by an Australian practitioner regulator (i.e. AHPRA), this may bias the outcome of the national-level scan in favour of AOPA, as a regulator in the same country. By describing the process used to select the core standards of AHPRA—noting they are highly regarded as best practice regulatory standards [23] in keeping with other similarly developed countries, such as the United Kingdom—we hope to have engendered confidence in the rigour of this approach. We acknowledge that the definition of *allied health practitioner* was adopted from the Australian context and may not be reflective of allied health as described by the International Standard Classification of Occupations (ISCO-08). Unfortunately, the ISCO-08 excludes orthotist/prosthetists—which are classified under Medical and Dental Prosthetic Technicians [49]—and as such, this international standard was not appropriate for the purpose of this scan.

This investigation describes AOPA’s regulatory journey as an example of an orthotic/prosthetic national association that has made the transition from limited to full self-regulation over several years. While many other organisations have also made this transition, we are unable to speak to the detail of their experiences with authenticity, and hope that the example of AOPA’s experience illuminates some of the barriers and facilitators to this transition.

## Conclusion

Establishing orthotist/prosthetist regulation is a valuable mechanism to ensure the delivery of safe and effective orthotic/prosthetic services, whilst simultaneously supporting workforce growth and increasing global access to these vital services. The overwhelming majority of orthotist/prosthetists are unregulated and, as such, the benefits of regulation are yet to be realised in many countries and globally. Increased regulatory support is required to remove barriers for national associations, particularly those in low-income countries with small workforces. An international body with the express purpose of supporting the development of national regulation for the orthotic/prosthetic profession will reduce barriers and increase global access to appropriate, safe and effective orthotic/prosthetic services for the growing number of people living with disability.

## Data Availability

All data generated or analysed during this study are included in this published article.

## Abbreviations

AMAS: Association Marocaine des Assistants et Assistantes Sociales (Social Work Association, Morocco)
AHPA: Allied Health Professions Australia
AHPRA: Australian Health Practitioner Regulation Agency
AOPA: Australian Orthotic Prosthetic Association
GAATO: Global Alliance of Assistive Technology Organisations
ISPO: International Society for Prosthetics and Orthotics
NASRHP: National Alliance of Self-Regulating Health Professions
WHO: World Health Organization
WCPT: World Confederation of Physical Therapy (now known as World Physiotherapy
